# Tissue plasminogen activator with prolonged dwell time effectively evacuates pleural effusions

**DOI:** 10.1186/s12890-022-02261-y

**Published:** 2022-12-05

**Authors:** Alexandra Townsend, Harsha Raju, Krystina A. Serpa, Rachel Pruett, Syed S. Razi, Francisco A. Tarrazzi, Catherine M. Tami, Mark I. Block

**Affiliations:** 1 Herbert Wertheim College of Medicine, Florida International University, Miami, FL 33199 USA; 2 Division of Thoracic Surgery, Memorial Healthcare System, 1150 N. 35th Ave., Suite 660, Hollywood, FL 33026 USA

**Keywords:** Parapneumonic effusion, Empyema, tPA, Fibrinolytic therapy

## Abstract

**Objectives:**

Fibrinolytic therapy can be effective for management of complex pleural effusions. Tissue plasminogen activator (tPA, 10 mg) and deoxyribonuclease (DNAse) every 12 h with a dwell time of one hour is a common strategy based on published data. We used a simpler protocol of tPA (4 mg) without DNAse but with a longer dwell time of 12 h, repeated daily. We reviewed our results.

**Methods:**

Charts were reviewed and demographics, clinical data and treatment information were abstracted. Outcomes were assessed based on radiographic findings and need for surgery.

**Results:**

Two hundred and fifteen effusions in 207 patients (8 bilateral) were identified. 85% were either infectious or malignant. Two hundred and forty nine chest tubes were used: 84% were 10 Fr or 12 Fr and 7% were PleurX®. Five hundred and thirty one doses of tPA were given. The median number of doses per effusion was 2 (range 1–10), and 84% of effusions were treated with three or fewer doses. There were no significant bleeding complications. Median time to chest tube removal was 6 days (range 1 to 98, IQR 4 to 10). Drainage was considered complete for 78% of effusions, while 6% required decortication.

**Conclusions:**

Low dose tPA daily with a 12 h dwell time may be as effective as the standard regimen of tPA and DNAse twice daily with one hour dwell. For most patients only three doses were required, and small pigtail catheters were sufficient. This regimen uses less medication and is logistically much easier than the current standard.

## Introduction

Intrapleural fibrinolytic therapy can be an effective alternative to decortication for the management of complex pleural effusions. Tillet and Sherry were the first to explore this strategy [1–3], reporting in 1949 and 1951 on the pharmacokinetics and effectiveness of streptokinase and streptococcal DNase. More recent prospective controlled trials of streptokinase [4–7] and then urokinase [8, 9] supported the potential utility of intrapleural fibrinolytic therapy, but fibrinolytic therapy did not gain widespread acceptance until publication of the Multi-Center Intrapleural Sepsis Trial 2 (MIST-2) in 2011 [10]. The MIST-2 used tissue plasminogen activator (tPA) [Genentech, San Francisco, CA, USA], an agent that was developed for use in treating coronary artery thrombosis and stroke. The use of tPA for intrapleural therapy was first documented in a case report by Walker and colleagues in 2003 [11].

MIST-2 reported that tPA and DNase reduced hospital length of stay and referral for surgery compared to placebo and to treatment with either agent alone. The agents were given with a dwell time of one hour, every 12 h for three days. This regimen can be cumbersome and logistically challenging because it calls for two drugs every 12 h with a short dwell time. In our early experience we transitioned to tPA alone and found that dwell times often inadvertently exceeded the prescribed one hour. We also found that it was difficult to adhere to an every-12 h dosing schedule. The serendipitous result was the discovery that tPA alone with a longer dwell time seemed effective. In a review of our experience treating 47 effusions in 46 patients with tPA alone, in doses ranging from 1 to 6 mg and dwell times ranging from 5 to 14 h, we found that only 6% required surgery [12]. Based on this review we concluded that a once daily dose of 4 mg with a dwell time of 12 h seemed most effective and most easily administered. Since then we have adhered to that regimen, and in this report we review that experience.

## Patients and methods

All methods were carried out in accordance with relevant guidelines and regulations in accordance with the Declaration of Helsinki.

Charts were reviewed for consecutive patients treated with tPA for complex pleural effusions between February 2018 and December 2020. Demographics and clinical data were collected from the electronic medical record (EMR).

### Chest tube placement and tPA therapy

A variety of catheters, including pigtail (8–14 Fr), standard chest tubes (24–28 Fr) and PleurX® [CareFusion, San Diego, CA, USA] catheters were used for drainage. When appropriate, image guidance with ultrasound or CT was used to facilitate accurate placement. Fibrinolytic therapy was considered as early as the next day if imaging following tube placement showed incomplete clearance of pleural opacity. Systemic anticoagulation was not considered a contraindication. Heparin drip therapy was held for four hours before and after administration of tPA. tPA (4 mg) in 50 mL saline was given through the chest tube at bedside and the chest tube was clamped for a target 12 h dwell time. The following day drainage was assessed and a determination was made about whether to repeat treatment. In general, tPA was repeated if drainage was more than 100 mL and not grossly bloody. Completeness of drainage was a subjective assessment by the treating physician based on imaging (chest x-ray and/or chest CT), considering factors such as evidence of trapped lung, residual pockets of fluid adjacent to the catheter that might resolve with additional treatment, or fluid in areas of the pleural space not adjacent to the catheter that might benefit from an additional image guided drain. There were no attempts to quantify residual fluid volume radiographically. Target therapy was for three doses over three days. If a residual loculated effusion separate from the drainage catheter was identified, an additional drain was placed and tPA therapy continued. Decortication was considered if the drainage was determined to be incomplete.

### Data collection and management

Data were abstracted from the EMR and stored in a REDCap database. These included patient demographics, pleural effusion analytics, drainage catheter size, duration and volume of drainage, dose and number of doses of tPA, serum hemoglobin, heparin drip therapy, transfusion therapy, length of hospital stay, and discharge status (alive/dead). Pre-treatment symptom duration was determined as the greater of the time from either subjective symptom onset or radiographic evidence of a pleural effusion to the first dose of tPA. Change in serum hemoglobin was determined by calculating the difference between the values from the blood draw closest to the start of treatment (but not more than three days before) and the blood draw at the latest of one to three days after the last dose of tPA.

### Outcome assessment

Outcomes were categorized into one of four groups based on imaging and medical record:

1. Complete drainage, defined as trace to no residual pleural fluid.
2. Incomplete drainage without surgery, defined as moderate residual pleural effusion but a clinical decision not to proceed to decortication.
3. Incomplete drainage with surgery.
4. Trapped lung, defined as incomplete expansion of the lung with a pneumothorax replacing the pleural effusion.

### Statistical analysis

A non-parametric Kruskal–Wallis ANOVA test was used to compare differences between symptom duration by outcome group.

## Results

### Patient characteristics

Review of medical records identified 207 patients with 215 effusions (eight patients had bilateral effusions) treated with at least one dose of tPA (Table 1). The average age was 63 years (range 20–96 years). Most patients were male and most effusions were right sided. The majority of effusions (64%) were classified as parapneumonic or empyema, and 20% were malignant. Heparin drip therapy was ongoing at the time of tPA therapy for 13 patients.

**Table 1:** Patient and Pleural Effusion Characteristics

Demographic	*N*	(%)
*Gender*		
Female	90	(43)
Male	117	(57)
*Laterality*		
Right	133	(62)
Left	82	(38)
*Etiology*		
Infectious	139	(65)
Malignant	44	(21)
CHF	2	(1)
Hepatic	3	(1)
Renal	5	(2)
Other	17	(8)
Unknown	5	(2)

### Chest tube use

Of the 215 effusions, 87% (*n* = 188) were managed with one chest tube. Two chest tubes were used for 20 effusions (9%) and three chest tubes were used for the remaining seven effusions (3%) for a total of 249 chest tubes. Chest tube sizes ranged from 8 to 28 Fr, but most were either 10 Fr (*n* = 149, 61%) or 12 Fr (*n* = 61, 25%).

### Fibrinolytic therapy

**Fig. 1:**
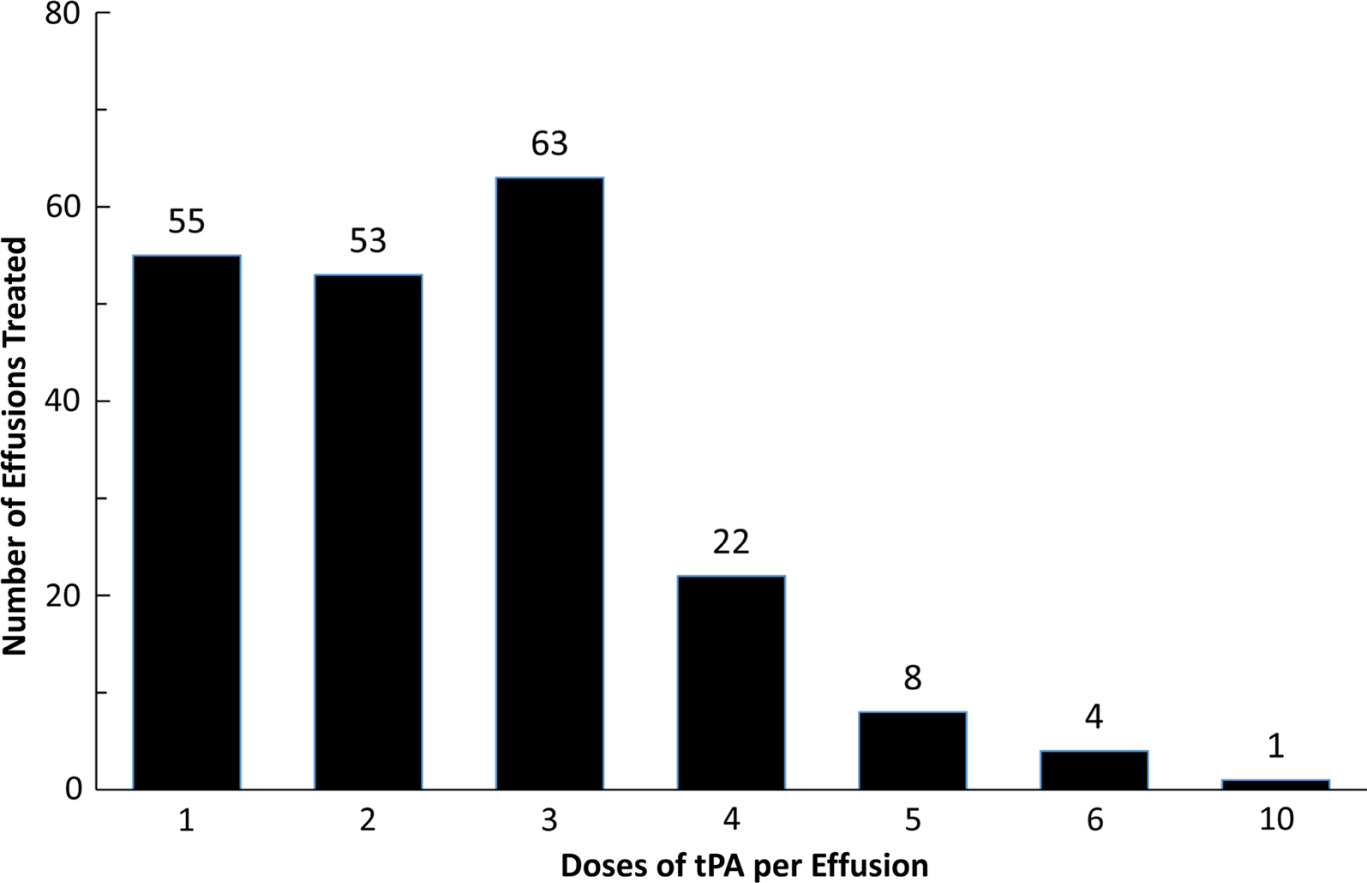
Number of Doses of tPA Given per Effusion. Note that 84% of effusions (180/215) were treated with three or fewer doses of tPA

**Fig. 2:**
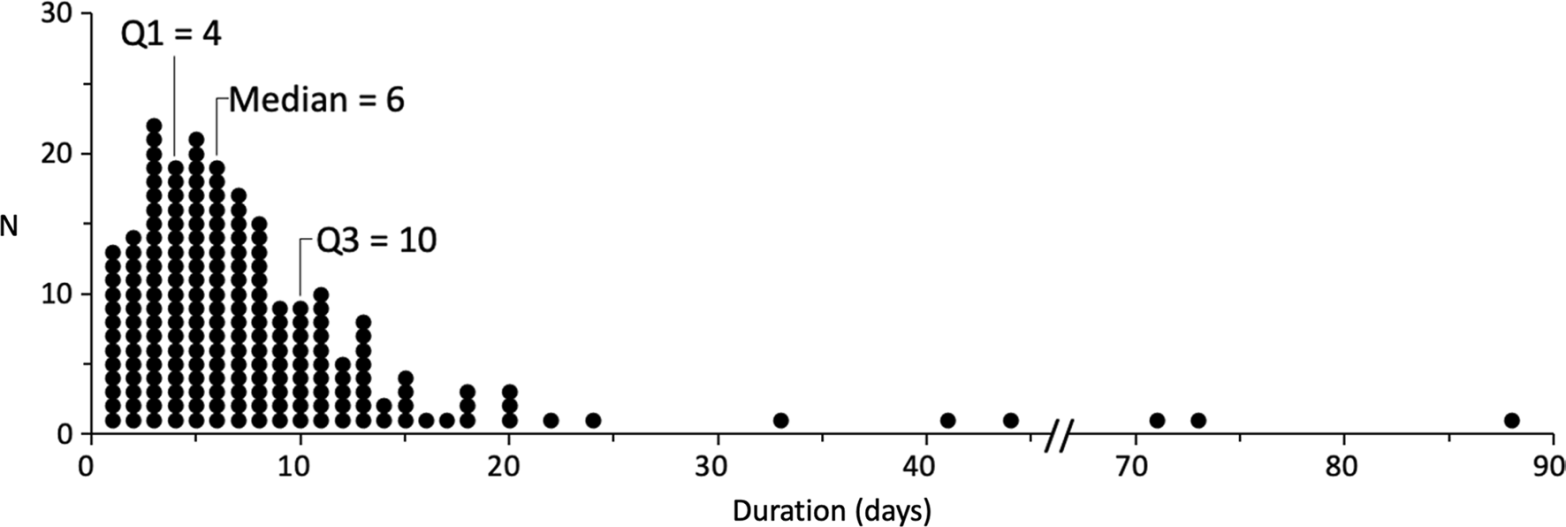
Duration of Therapy. Duration of therapy was determined as the number of days from the first dose of tPA to the day the chest tube was removed. Median = 6 days, IQR (Q1:Q3) = 4 to 10 days

512 doses of tPA were given. Though doses ranged from 2 to 6 mg, 91% were 4 mg. Dwell times ranged from 2 to 21 h, with the target of 12 h achieved for 98% of doses. On eight occasions the tubes were unclamped before the target dwell time. The number of doses per effusion ranged from 1 to 10 (median = 2) with 84% of effusions treated with the target regimen of three or fewer doses (Fig. 1). Duration of chest tube drainage measured from the first dose of tPA to when the chest tube was removed ranged from 1 to 98 days, with a median of 6 days (IQR: 4 to 10 days; Fig. 2). The values for volume of chest tube drainage that were extracted from the nursing record and progress notes were inconsistent and therefore considered unreliable and are not reported.

### Bleeding complications

**Fig. 3:**
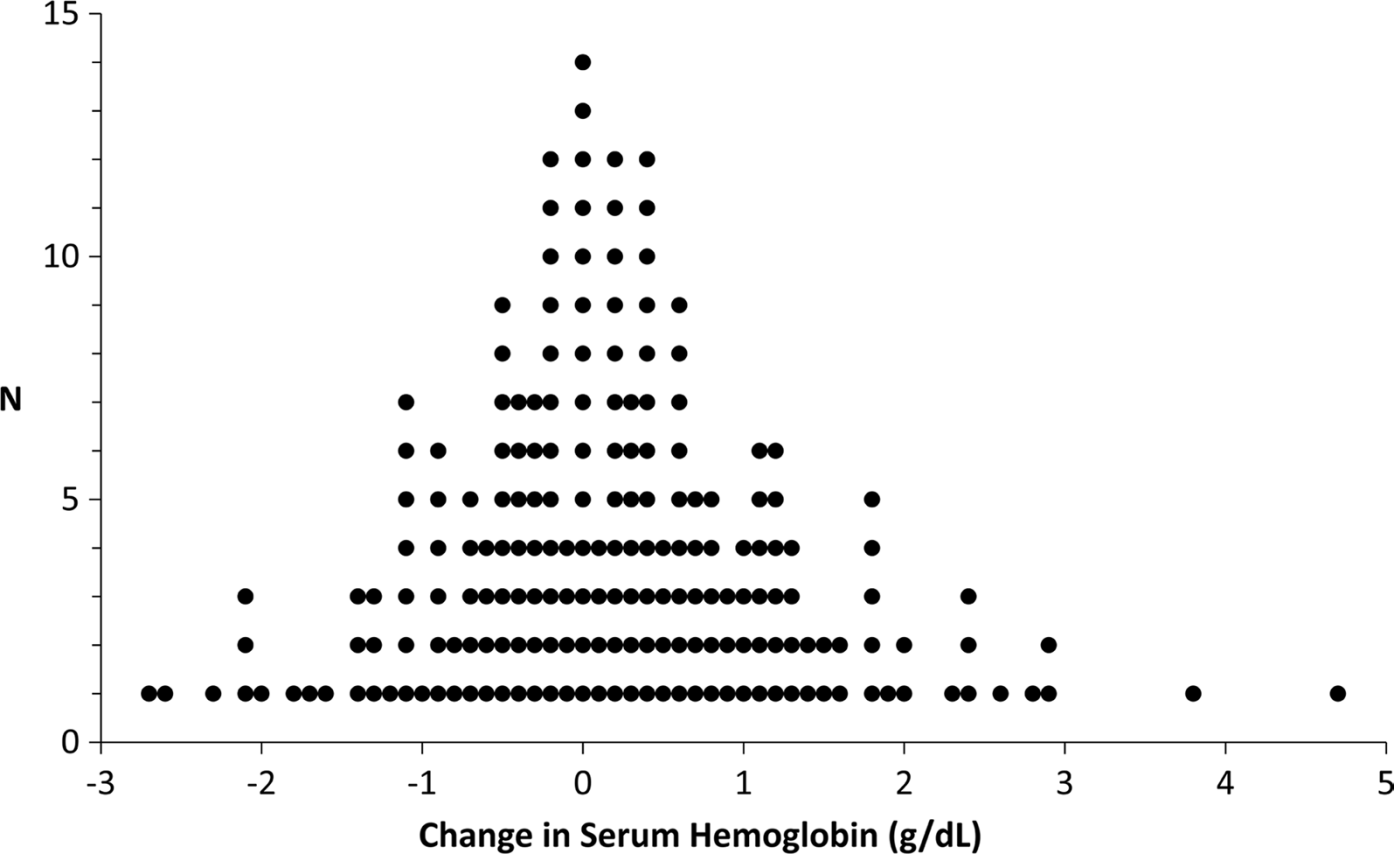
Change in Serum Hemoglobin. Frequency distribution plot of change in serum hemoglobin with tPA therapy, measured as the difference between the values from the blood draw closest to the start of treatment (but not more than three days before) and the blood draw at the latest of one to three days after the last dose of tPA. Data were available for 201/207 patients

**Fig. 4:**
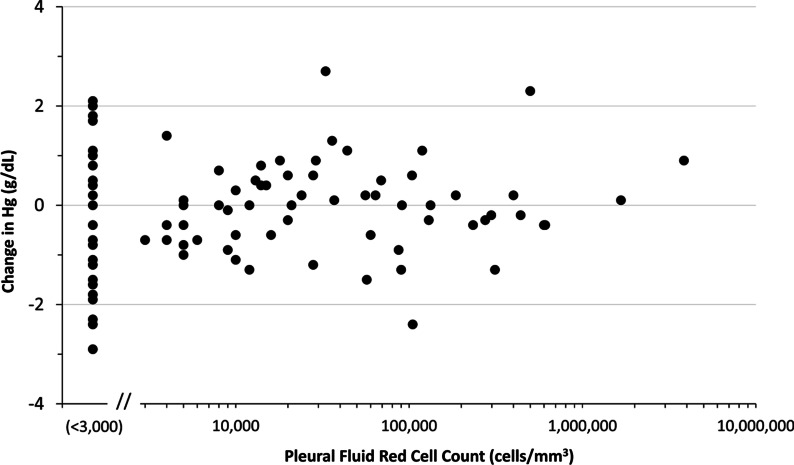
Change in Serum Hemoglobin by Pleural Fluid Red Cell Count. Both pleural fluid cell count and serum hemoglobin before and after tPA therapy were available for 95 patients. Of these, pleural fluid red cell count was reported as “ < 3,000 cells/mm^3^” for 35

No patient experienced hemodynamically significant bleeding as defined by a requirement for alteration in level of care or initiation of intravenous resuscitation. In one patient tPA therapy was not continued because chest tube drainage turned grossly bloody. Change in serum hemoglobin levels could be determined in 201 patients and ranged from a decline of 2.7 g/dL to a rise of 4.7 g/dL (median =  + 0.2 g/dL, IQR = − 0.5 g/dL to + 0.8 g/dL; Fig. 3). For 95 patients both pleural fluid red cell count and change in serum hemoglobin were available, and no correlation was evident (Fig. 4). Twelve patients (5.6%) received a blood transfusion during or within three days of completing tPA therapy.

### Outcome

**Fig. 5:**
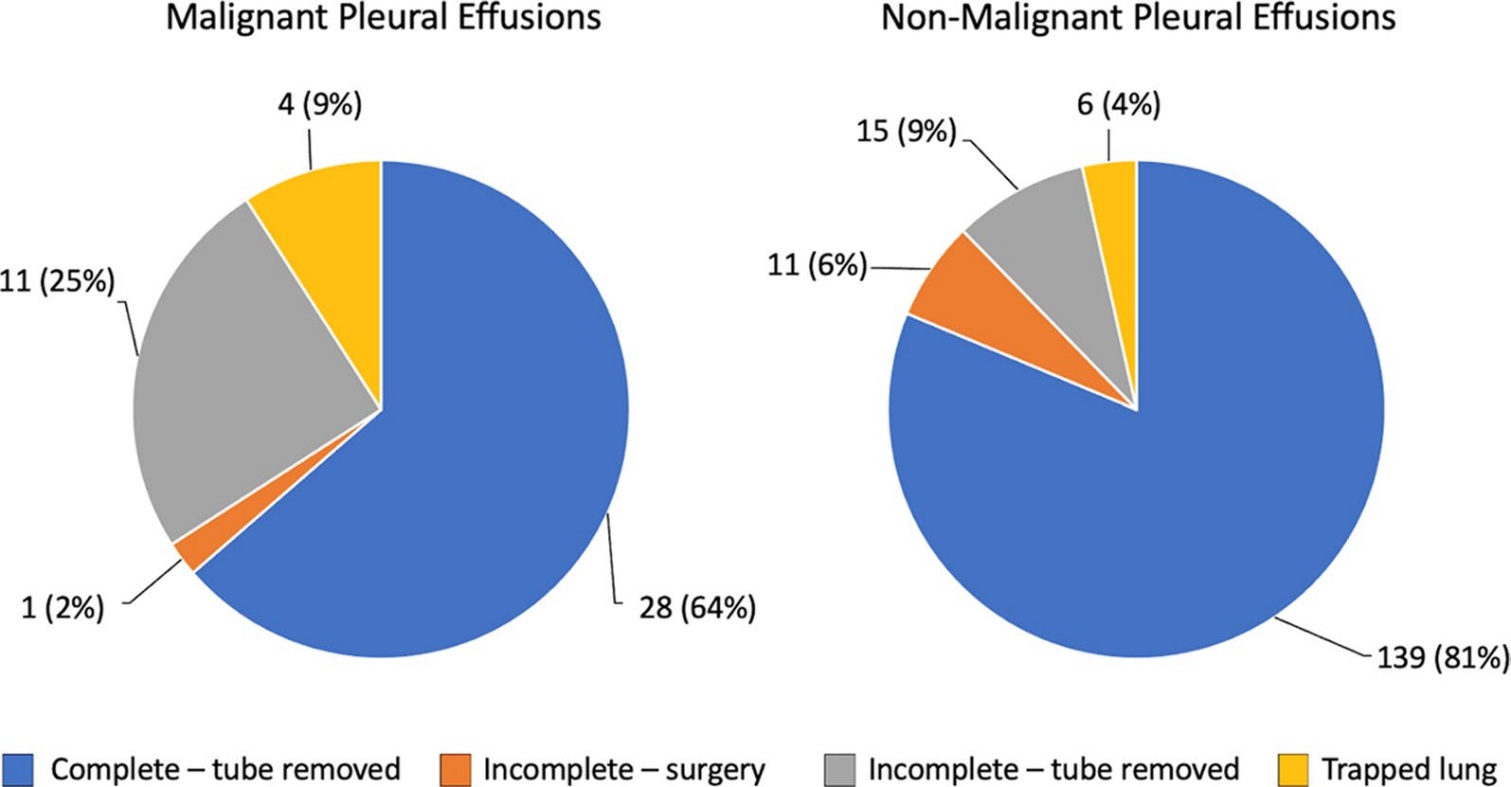
Outcome Stratified by Malignant and Non-Malignant Effusions. Outcomes were classified into one of four categories based on radiographic findings and decision for surgery (see Methods)

**Fig. 6:**
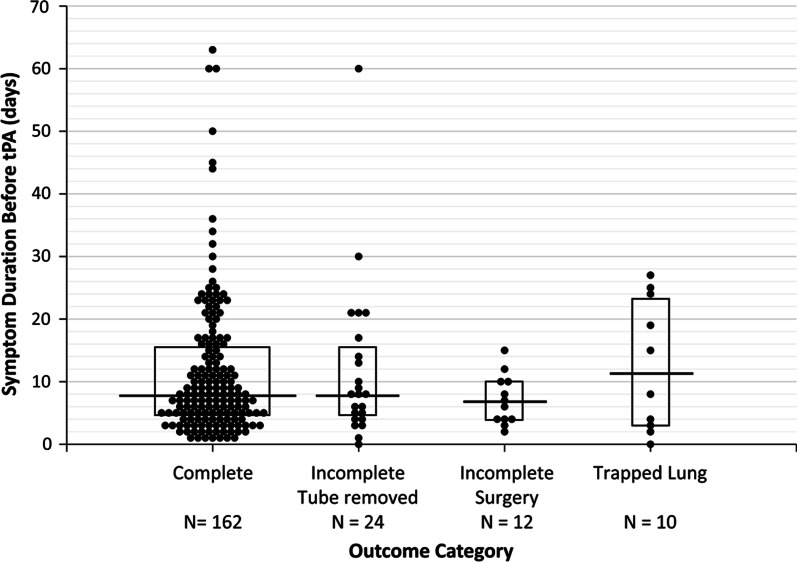
Outcome by Symptom Duration. Symptom duration grouped according to patient outcome. Horizontal bars indicate group median; boxes indicate the interquartile range

Most effusions (*n* = 167, 77.7%) were assessed to be completely drained and required no additional interventions. Of the remaining 48, 26 (12.1%) had incomplete drainage but were assessed to require no further therapy, 12 (5.6%) underwent decortication and 10 (4.7%) were diagnosed with trapped lung. When stratified by etiology, malignant effusions were less likely than non-malignant effusions to be completely drained with tPA therapy, although complete drainage was still achieved in 64% of cases (Fig. 5). When stratified by chest tube size, the outcome of complete drainage was seen in approximately 80% of effusions across all chest tube sizes (Table 2). Symptom duration was discernable in 208 patients and ranged from 0 to 63 days (median = 8 days, IQR = 4–15 days). When stratified by outcome, median symptom duration was shortest in the group that required surgery (6.5 days) and longest in the group with trapped lung (11.5 days). Median duration was 8 days in both of the other two groups (Fig. 6). These differences were not statistically significant (*p* = 0.70). We also investigated the potential relationships between outcome and pleural fluid pH, LDH, protein and white cell count but found no correlation (data not shown).

**Table 2:** Outcome Stratified by Chest Tube Size

Largest tube size	Complete–tube removed		Incomplete–surgery	Incomplete–tube removed, no further therapy
	*N*	(%)		
8 Fr	2	(100)	0	0
10 Fr	96	(83)	5	15
12 Fr	47	(82)	3	7
14 Fr	5	(60)	2	1
16 Fr	1		0	1
24 Fr	1	(80)	0	0
28 Fr	3		1	0
PleurX®	12	(80)	1	2

## Discussion

Tissue plasminogen activator was developed for the treatment of coronary artery thrombosis and stroke, and its use for intrapleural therapy was first documented in a case report by Walker and colleagues in 2003 [11]. Subsequent studies supported its safety and potential efficacy [13, 14], but it was the MIST-2, published in 2011 [10], that led to the general adoption of tPA and DNase for management of complex effusions. The MIST-2 was a multi-center prospective randomized trial that enrolled 210 patients and compared the combination of tPA and DNAse to placebo and to either agent alone. The primary outcome of change in pleural opacity was greatest in the tPA + DNase group. The secondary outcome of referral for surgery was lowest in the tPA + DNase group (4%), which was statistically significantly lower than in the placebo (16%, *p* = 0.03) and DNase alone groups (39%, *p* = 0.01), but was not significantly lower than in the tPA alone group (6%, *p* = 0.10). In attempting to replicate the protocol for tPA + DNase in our patients we encountered logistical challenges with using both agents simultaneously, and in delivering the doses every 12 h. Furthermore, we discovered that chest tubes often were not unclamped for several hours beyond the prescribed one hour dwell time. In these patients effusion drainage was surprisingly brisk, leading us to modify our treatment regimen to the use of tPA alone with a prolonged dwell time. In reviewing that early experience we concluded that a 4 mg dose of tPA was sufficient, and that a 12 h dwell time was most effective [12]. In this report we review our experience with that standardized protocol.

The findings in the current report support the initial clinical impression that our modified protocol is effective in the non-surgical management of complex pleural effusions. Some patients were discovered to have trapped lung, a condition that requires surgical decortication and that would not reasonably be expected to respond to fibrinolytic therapy. In our experience, tPA therapy led to prompt diagnosis of trapped lung and facilitated appropriate decision-making regarding the indications for and timing of decortication. Of the patients without trapped lung, for whom fibrinolytic therapy has the potential to be definitive therapy, 81% were considered to have complete drainage with no further intervention indicated, and only 6% underwent surgery to clear residual loculated effusion. Although not a prospective randomized trial, this result is nearly identical to the results reported in the tPA + DNase and tPA alone groups in MIST-2.

Bleeding is a concern with intrapleural tPA. In this series no patient experienced life-threatening hemorrhage and only one patient had treatment discontinued because of grossly bloody chest tube drainage; 5.6% of patients were given a transfusion. We calculated change in serum hemoglobin from before to after tPA treatment as a surrogate measure of blood loss and found that results resembled a normal distribution around 0. This suggests that intrapleural tPA did not consistently contribute to blood loss. Based on our earlier experience, and confirmed in this series, systemic anticoagulation is not a contraindication to intrapleural tPA, and 13 patients in this series were receiving heparin drips at the time of therapy. Even with bloody pleural effusions, especially in the setting of a malignant pleural effusion, tPA can be given without causing significant hemorrhage. Indeed, in the 95 patients for whom pleural fluid red cell count was available, higher counts were not associated with a greater drop in hemoglobin. It has been our experience that tPA for bloody, malignant effusions is often very helpful because it promotes complete drainage and pleural apposition that tamponades further bleeding.

A related concern with intrapleural tPA is the potential for systemic effects. In no patient was coagulopathic bleeding noted at sites such as intravenous lines or drains to suggest systemic effects, and the known pharmacokinetics of tPA suggests this should not be a concern. We are not aware of any data that intrapleural tPA is absorbed into the systemic circulation to any significant degree, and given that the first-pass half-life of tPA in serum is less than five minutes [15–17], it reasons that any tPA absorbed across the pleura would be rapidly metabolized. It seems therefore highly unlikely that intrapleural tPA poses a significant risk of systemic hemorrhage.

Because a large proportion of our patients were treated with small, “pigtail” catheters, we were able to also evaluate the efficacy of small bore catheters. The optimal choice of tube size to facilitate effective drainage with fibrinolytics is a topic of some controversy. Intuitively a larger tube should be less likely to clog and therefore facilitate more effective drainage, yet smaller tubes are better tolerated and more easily targeted to specific areas with image guidance. In a separately published analysis of the MIST-1 data, in which tube sizes ranged from < 10 Fr to > 20 Fr, Rahman and colleagues reported that tube size had no effect on outcome [18]. Our results support this conclusion.

A concern with embarking on a course of fibrinolytic therapy rather than proceeding immediately to surgery is that it can prolong hospitalization. In our experience, half of patients completed therapy within six days. Because a handful of patients had extremely prolonged chest tube drainage, mean duration of chest tube drainage in our study was 8.5 days. The MIST-2 did not report duration of chest tube drainage, but mean length of hospitalization in the tPA + DNAase group, which was statistically significantly shorter than for all other groups, was 11.8 ± 9.4 days when prolonged-stay outliers were discarded. This suggests that our protocol compares favorably to the standardized MIST-2 regimen. Furthermore, we believe this number can be reduced with consistent implementation of a care-algorithm such that length-of-stay can be similar to, and perhaps even shorter than surgery.

Objective assessment of outcome is a significant challenge in evaluating the effectiveness of various strategies for managing complex pleural effusions. Existing studies of fibrinolytic therapy have used a variety of outcome metrics, including length of hospital stay, all-cause mortality, need for surgery, or radiographic improvement. All of these metrics have a significant subjective component. The MIST-2 used reduction in pleural opacity (on chest x-ray) as a primary endpoint, and referral for surgery as a secondary endpoint. Although imaging algorithms can provide objectivity to the radiographic analysis of chest x-ray findings, they may not be able to accurately distinguish between pleural fluid, atelectasis and consolidation as the cause of radiographic opacity. Furthermore, although reduction in radiographic opacity may reflect efficacy of therapy, ultimately the outcome that matters is the volume of residual effusion, not how much smaller it is from where it started. The need for surgery is a highly subjective assessment by the treating team, and therefore has its limitations as an objective outcome measure as well. In our study, we used the combination of radiographic assessment and the decision for surgery as an outcome measure. While this should provide some degree of comparability to results of other studies, its highly subjective nature makes firm conclusions suspect and direct comparison to alternative strategies difficult. High-resolution imaging with chest CT, especially when done with intravenous contrast, is ideally suited for distinguishing pleural fluid from adjacent atelectasis and consolidation, and software algorithms can be constructed to automatically calculate volumes. However, frequent and repeated use of chest CT to evaluate therapeutic progress with pleural effusions can expose the patient to potentially high levels of radiation. Development of a high-resolution volumetric measure that minimizes radiation exposure would be very helpful for objective assessment of fibrinolytic efficacy.

The half-life of tPA in the blood is minutes, but there is no information on its half-life in pleural fluid. tPA is a naturally occurring protein in the pleural space and so it is reasonable that the half-life is prolonged. Furthermore, in a study of tPA in cerebrospinal fluid Kramer and colleagues [19] found that concentrations remained elevated for six and, in a few patients, even 12 h after a single dose. In our experience with a 12-h dwell time occasionally patients would complain of increasing chest pressure beginning about six hours after the administration of the tPA. This experience, and the observation that a dwell time of up to 12 h can be highly effective, suggest that the half-life of tPA in the pleural space may be several hours.

There are several limitations to this study. As a retrospective chart review the results are subject to selection bias and there may have been bias in clinical decision-making that subsequently affected the determination of outcome measures. However it is a consecutive series of patients and the study population is large, so that although the results may not be directly comparable to results from other studies because of these biases, the primary conclusion remains provocative. Another limitation is that we have not included longer-term follow-up to confirm that effusions considered effectively treated did not recur. Our use of change in serum hemoglobin to evaluate bleeding incidence is questionable because of multiple uncontrolled and confounding variables.

There are two significant advantages of our regimen compared to MIST-2. First, our protocol is significantly easier to implement. In an environment where rules prohibit nurses from administering medication through chest tubes, and where 24-h physician coverage is limited, it is much easier to adhere to a single dose daily than to a dose every 12 h. And secondly, because we used a lower dose of tPA and did not use DNase, the regimen is less costly. According to our pharmacy, the Wholesale Acquisition Cost for Pulmozyme® (DNAse) is $111.47 per 1 mg, and for Activase® (tPA) is $148.47 per 2 mg. Therefore the drug costs alone for one day of the MIST-2 regimen (Pulmozyme® 5 mg and Activase® 10 mg twice daily) is $2,599.40 compared to $296.84 for one day of our regimen.

The natural history of pleural fibrosis suggests that symptom duration, and perhaps other clinical features should predict the efficacy of intrapleural fibrinolytic therapy. However symptom duration is a highly subjective and unreliable metric, and proved essentially useless as a predictor of outcome in our series. Pleural fluid characteristics also had no predictive value. As a result it is our practice to give tPA if imaging shows incomplete pleural drainage regardless of effusion etiology or duration. Minimal drainage (< 100 mL) or imaging showing a trapped lung indicates that further tPA therapy is futile, while a high volume of drainage suggests that further therapy may be successful. This protocol facilitates rapid clinical decision-making and is safe.

## Conclusion

Our findings suggest that a low dose of tPA with an extended dwell time given daily was effective in the management of complex pleural effusions. This protocol is more practical and less expensive than the combination of tPA and DNAse given twice daily, and therefore more likely to be widely adopted. Our results suggest several future directions. A randomized controlled trial to compare our protocol to MIST-2 is planned, as well as development and validation of a modified low-dose chest CT protocol for objective measurement of effusion volume. Additionally, pharmacokinetic studies to determine the half-life of tPA in pleural fluid are underway.

## Data Availability

The datasets used and analysed during this study are available from the corresponding author on reasonable request.

## Abbreviations

tPA: Tissue plasminogen activator
DNase: Deoxyribonuclease
MIST-2: Multi-Center Intrapleural Sepsis Trial 2
EMR: Electronic Medical Record
REDCap: Research Electronic Data Capture
Fr: French size, catheter
CT: Computer Tomography
ANOVA: Analysis of Variance
IQR: Interquartile range
LDH: Lactate dehydrogenase
